# Long Term Impact on Lung Function of Patients With Moderate and Severe COVID-19. A Prospective Cohort Study

**DOI:** 10.3389/fpubh.2021.663076

**Published:** 2021-12-07

**Authors:** Sonia Qureshi, Nosheen Nasir, Naveed Rashid, Naveed Ahmed, Zoya Haq, Farah Qamar

**Affiliations:** ^1^Departments of Pediatrics and Child Health, Aga Khan University, Karachi, Pakistan; ^2^Department of Medicine, The Aga Khan University, Karachi, Pakistan; ^3^Liaquat National Medical College, Karachi, Pakistan

**Keywords:** long-term impact, lung function, COVID-19, spirometry, DLCO

## Abstract

**Background:** In Pakistan, the cases of COVID-19 have declined from 6000 per day in June to 600 in September 2020. A significant number of patients continue to recover from the disease, however, little is known about the lung function capacity among survivors. We aim to determine the long-term impact on lung function capacity in patients who have survived moderate or severe COVID-19 disease in a resource-poor setting.

**Methods:** This prospective cohort study will be conducted at Aga Khan University Hospital (AKUH), Karachi Pakistan. Patients 15 years and above who have survived an episode of moderate or severe COVID-19, have reverse transcriptase-polymerase chain reaction (RT-PCR) positive for COVID 19 (nasopharyngeal or oropharyngeal) will be included. Patients with a pre-existing diagnosis of obstructive or interstitial lung disease, lung fibrosis, lung cancers, connective tissue disorders, autoimmune conditions affecting the lungs, underlying heart disease, history of syncope and those who refuse to participate will be excluded from the study. Pulmonary function will be assessed using spirometry and diffusion lung capacity for carbon monoxide (DLCO) at 3- and 6-months interval from the time of discharge from the hospital. Additionally, a chest X-ray and CT-chest will be performed if clinically indicated after consultation with the study pulmonologist or Infectious Disease (ID) physician. Echocardiogram (ECHO) will be performed to look for pulmonary hypertension at the 3 month visit and repeated at 6 months in case any abnormality is identified in the initial ECHO. Data analysis will be performed using standard statistical software. The study was approved by the Ethical Review Committee (ERC) of the institution (ERC reference number 2020-4735-11311).

**Strengths and Limitations of the Study:** This cohort study will provide evidence on the long-term impact on lung function among COVID-19 survivors with moderate to severe disease. Such data will be key in understanding the impact of the disease on vital functions and will help devise rehabilitative strategies to best overcome the effects of disease. However, this will be a single-center, study recruiting only a limited number of COVID-19 survivors.

## Background

The severe acute respiratory syndrome coronavirus 2 (SARS-CoV-2) also known as COVID-19, was declared as a global pandemic by World Health Organization (WHO) during first quarter of 2020. The virus was traced to the Chinese province of Wuhan where it first emerged in December 2019 (1). Globally, as of November 7, 2020, there are 49,685,311 confirmed cases of COVID-19, including 1,249,030 deaths, reported to WHO (2). The case fatality rate of COVID-19 is lower (2%) in contrast, Severe Acute Respiratory Syndrome (SARS) in 2003 (~10% CFR) and Middle East respiratory syndrome coronavirus (MERS-CoV) in 2012 (34% CFR) (3, 4). In Pakistan, after implementation of a 6-month, nation-wide lockdown the number of cases declines, with daily active cases being 666, on 12th October 2020, as compared to the peak of 6,825, during June 2020 (5). However, a second wave of COVID-19 cases is now being observed throughout the country which is believed to be due to non-compliance of standard operating procedures (SOPs) imposed by the government (6).

Since COVID-19 has a predilection for the lung, concerns have been raised about the restoration of lung function after apparent recovery from the disease. COVID-19 presents with a variable degree of clinical severity, ranging from mild upper respiratory tract illness to severe interstitial pneumonia and acute respiratory distress syndrome (ARDS) (7–9). MERS, Intensive Care Unit (ICU) survivors have been reported to have limitations in some measures which altered their quality of life and led to long term psychological morbidities when compared to patients with less severe illness managed in medical wards (10). The pathophysiological features of severe Covid-19 are dominated by an acute pneumonic process with an extensive radiologic opacity and, on autopsy, diffuse alveolar damage, inflammation, microvascular thrombosis along with capillary congestion in lungs (10). Recovered and discharged patients with COVID-19 pneumonia were seen to have residual abnormalities in chest computed tomography (CT) scans, with ground-glass opacity as the most common pattern (11).

Older patients with co-morbidities such as hypertension, diabetes, malignancies and risk factors such as smoking were found to be at greater risk of severe disease. Comorbidities have been correlated with poorer clinical outcomes (12). In addition to the direct effects of the disease, increased medical cost and decreased capacity to generate income due to severe, prolonged illness might be cumbersome for families. Considering the novelty and unpredictability of the disease, it is critical to study the long-term impact on lung function of patients recovered from the disease.

## Aim

To monitor the lung function of patients with moderate and severe COVID-19 disease at 3 and 6 months by performing pulmonary function test at a tertiary care hospital of a low-middle income country.

## Research Question

What is the long-term impact of moderate or severe COVID-19 illness on pulmonary functions?

## Research Objective

To determine the long-term impact on lung function in patients suffering from moderate and severe COVID-19 disease in a low- middle income country.

## Methods

### Study Design and Setting

This prospective cohort study will be undertaken at Aga Khan University Hospital (AKUH) Karachi, Pakistan. AKUH is one of the tertiary care hospitals in Karachi Pakistan with inpatient bed capacity more than 700 and a 24/7 operational emergency room. It consists of undergraduate medical and nursing school, and postgraduate training programs. In terms of services, the institute has advanced capacity in almost every specialty such as internal medicine, infectious disease, hematology, and pathology and laboratory services. It is one of the four institutes across Pakistan to initiate COVID-19 testing early in the outbreak and reached to testing capacity of almost >1000 tests per day. AKUH led the training and guideline development for management of COVID-19 in Pakistan.

### Study Population

All patients aged 15 years and above with a PCR confirmed diagnosis of COVID-19, having moderate or severe COVID-19 disease as per WHO criteria with a pre-defined inclusion and exclusion criteria will be enrolled in the study (13).

### Inclusion Criteria

1. Patients of age ≥15 years who suffered from moderate or severe illness related to COVID-19 during an outbreak (classified using WHO criteria).
2. RT-PCR positive for COVID 19 (Nasopharyngeal or oropharyngeal).
3. Consent to participate.

### Exclusion Criteria

1. Patients suffering from underlying obstructive disease such as asthma, chronic obstructive pulmonary disease (COPD), bronchiectasis or interstitial lung disease, lung fibrosis, lung cancers, connective tissue disorders like scleroderma, autoimmune conditions affecting the lungs like sarcoidosis and Wegener's disease.
2. Patients with underlying heart disease.
3. Patients with history of syncope.

### Sampling Technique

Patients suffering from moderate or severe COVID-19 with positive nasopharyngeal or oropharyngeal RT-PCR will be recruited consecutively from the study site. COVID-19 patients admitted in the ICU or high dependency unit (HDU) will be screened for inclusion in the study. Eligible patients will be approached for inclusion in the study after discharge from the hospital. Participants will be contacted over phone within 1 month of discharge from hospital and verbal consent will be obtained.

Once the consent has been obtained, an appointment for pulmonary function test (spirometry/DLCO), chest x-ray and echocardiogram (ECHO) will be scheduled at third month post discharge (first follow up). In order to facilitate the patients and avoid overflow in clinical areas, the appointment will be kept flexible with 1-week (before or after) window according to availability of patient and appointment slot.

**Figure 1:**
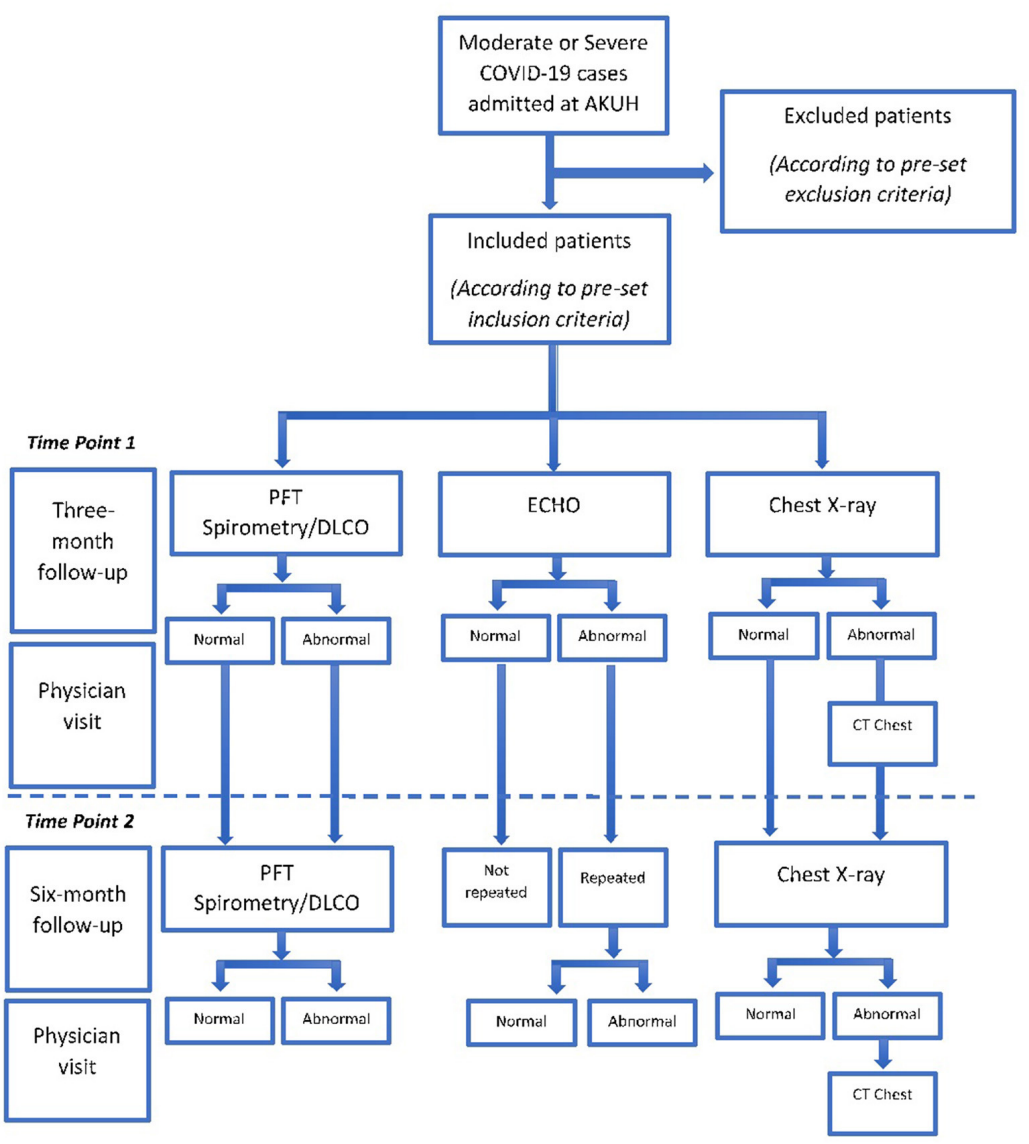
Study flow chart: time points of follow-ups, testing, and physician visits.

Following the investigations, the patients will be asked to visit for consultation with a pulmonologist or infectious disease consultant to discuss the reports and further advise on the disease. Patients will be thoroughly examined by the consultant and advised further radiology examination if needed. A second follow up will be scheduled at 6 months for a repeat, pulmonary function test and chest X-ray. ECHO will be repeated only if an abnormality is reported at the 3 month follow up. A 6-month follow-up visit with consultant will be scheduled to predict the outcome of the disease (Figure 1). CT chest will be performed at 3-month follow-up in patients whose chest X-ray shows persistent abnormality when compared to the baseline shows a new abnormality or on the physicians' recommendation.

### Sample Size

This will be an exploratory study therefore; we will enroll 60–70 confirmed COVID-19 cases fulfilling the inclusion criteria. Most (80%) of COVID-19 cases are mild, and the number of COVID-19 cases is expected to decline in the next 3 months thus, an arbitrary sample size of 60–70 is selected for convenience and feasibility. This sample may be underpowered to detect the outcome however; it will be helpful in generating the proof of principle.

### Data Collection Tool

All clinical, epidemiological, demographic, and laboratory data will be collected on a predesigned paper-based questionnaire. The questionnaire consists of sections for demographics, clinical and laboratory data. The physician notes will be extracted from the medical record file of the patients. Following study tools will be used to collect data;

1. Screening form for evaluation of eligibility of participants.
2. Questionnaire to record demographics, date of onset of disease, medical history including comorbidities, smoking status, influenza vaccine history, travel history. The information on this form will be collected from a number of sources including patients, parents/caregivers and hospital records.
3. Laboratory form will be used to record findings of lung function test, Chest X-ray, CT-chest and ECHO findings at 3 and 6 months follow-ups.

### Data Collection Procedure

The data will be collected from different sources and time points from diagnosis to final follow-up of the study as depicted in Table 1. Lung function impairment will be measured by performing pulmonary function test (spirometry and DLCO) and reported as a categorical variable, as defined by the guidelines of the American Thoracic Society (ATS). COVID-19 disease will be categorized as moderate or severe/critical illness based on World Health Organization (WHO) Criteria depicted in Table 2.

**Table 1:** Study variables and time points for data collection.

**S.no**	**Variables**	**Hospital admission records**				**Prospective from patient records**	
		**Baseline**	**24–48 h Pre-administration of anti-COVID-19 medication**	**24–48 h Post-administration of anti-COVID-19 medication**	^**^At the time of discharge	**Three-month follow-up**	**Six-month follow-up**
1	Age (years)	x					
2	Gender	x					
3	Weight (kg)	x					
4	Height (cm)	x					
5	Smoking status	x					
6	Date of onset of illness	x					
7	Fever at the onset of illness	x					
8	Difficulty in breathing at the onset of illness	x					
9	Duration of illness (days)	x					
10	Co-morbids	x					
11	^***^CURB-65 score during hospital stay	x					
12	^***^qSOFA score during hospital stay	x					
13	ICU stay	x					
14	Length of stay at ICU	x					
15	Length of stay at hospital	x					
16	Travel history	x^*^				x^*^	
17	History of influenza vaccine	x^*^				x^*^	
18	Treatment with anti-COVID-19 medicines	x					
19	Day of illness at which RT-PCR negative	x					
20	Need of mechanical ventilation	x					
21	Laboratory investigations	x	x	x	x	x	x
22	Radiology (CXR and/or CT-Chest)	x	x	x	x	x	x
23	DLCO/Spirometry					x	x
24	ECHO				x	x	x

* *If not available in hospital records*.

** *Laboratory and radiology tests will be considered as baseline for further reference in the follow-ups*.

*** *CURB-65: for assessing pneumonia severity, it ranges from 0 to 5. CURB-score ≥ 3 labeled as severe pneumonia. qSOFA score: Quick Sequential Organ Failure Assessment score, it ranges from 0 to 3 points. The presence of two or more qSOFA points near the onset of infection is associated with a greater risk of death or prolonged intensive care unit stay*.

**Table 2:** World Health Organization (WHO)–COVID-19 disease classification.

**COVID-19 disease category**	**Children**	**Adults**
^*^Moderate	Clinical signs of non-severe pneumonia (cough or difficulty breathing + fast breathing and/or chest indrawing) and no signs of severe pneumonia. Fast breathing (in breaths/min): <2 months: RR ≥60 2–11 months: RR ≥ 50 1–5 years: RR ≥ 40	Clinical signs of pneumonia (fever, cough, dyspnoea, fast breathing) but no signs of severe pneumonia, including SpO2 > 90% on room air.
^*^Severe	Clinical signs of pneumonia (cough or difficulty in breathing) + at least one of the following: Central cyanosis or SpO2 <90%; severe respiratory distress (e.g., fast breathing, grunting, very severe chest indrawing); general danger sign: inability to breastfeed or drink, lethargy or unconsciousness, or convulsions Fast breathing (in breaths/min): <2 months: RR ≥ 60 2–11 months: RR ≥ 50 1–5 years: RR ≥ 40	Clinical signs of pneumonia (fever, cough, dyspnoea, fast breathing) plus one of the following: Respiratory rate > 30 breaths/min; Severe respiratory distress; SpO2 <90% on room air

* *While the diagnosis can be made on clinical grounds; chest imaging (radiograph, CT scan, and ultrasound) may assist in diagnosis and identify or exclude pulmonary complications*.

*Source: World Health Organization (13)*.

Medical record files of the patients will be reviewed to extract the data on diagnosis and management throughout the hospital stay. Demographic data, presenting complaints, co-morbidities, smoking and travel history will be recorded one time. Moreover, quick Sequential Organ Failure Assessment (qSOFA) and (CURB-65) scores will also be calculated anytime during hospital stay. Conversely, the radiology and laboratory data of 24–72 h before and after the administration of anti- COVID-19 disease medications (remdesivir, hydroxychloroquine, convalescent Plasma therapy etc.) and steroids will be mentioned. Any missing information from medical record file will be collected directly from patient at the time of follow-up.

### Planning and Training

Necessary approvals from ethical review committees will be taken. Training will be conducted for study staff at the study site (AKUH) by the study investigators. The training will focus on introduction of the project and the team members, administration and completeness of data collection tools including informed consent, communication skills and other study activities. The staff will also be trained on his/her own safety during this outbreak and training on usage of personal protective equipment will also be provided.

### Data Management, Storage, and Security

All data will be collected at the study site using the data collection tool specifically developed for the study. Each participant will be assigned unique study identification. Data will be collected on paper-based forms, secured in a locked cabinet at the study site. Data will be entered in SPSS and stored in password protected-user specific computer database. Only the study investigators will have access to the data. If the data entry error rate is >10%, the data will be checked. In case when the error rate is <10%, discrepant entries will be verified and corrected. Daily Log sheet will be maintained by the study staff for screening and enrolling the participants and to maintain their follow-ups. The study staff will maintain a log sheet for performing and recording the lung function test, CXR, ECHO and CT-chest findings.

### Ethical Consideration

The project was approved by the Ethical Review Committee (ERC) of the AKUH (ERC reference number 2020-4735-11311).

### Consent and Assent

Informed consent will be taken from patients, parents, or legal guardians of the child. Purpose of the study as well as all study procedures will be explained in detail to the caregiver/participant. Assent will also be obtained from the children of age 15 years-18 years in addition to consent from their legal caregiver/parents. Verbal consent will be obtained on phone at least 2 weeks after hospital discharge if fulfills the eligibility criteria. Written informed consent/assent will be obtained at 3-month visit to a hospital for testing. The process of obtaining consent may take up to 15–20 min.

### Risks and Benefits

There are no risks in participating in this study. In rare cases, patient may experience light-headedness/syncope during the pulmonary function test. Patients participating in this study will have an access to their investigations to track the progress of their lung functions. Patients will be followed up in the clinic and examined by an ID physician and/or pulmonologist free of cost. All the tests which are part of the study protocol will be performed from the study budget. The study may cover the travel to and from the hospital for participants needing support. No other direct benefits to the individual participant are anticipated.

### Plan of Statistical Analysis

The data will be analyzed using standard statistical software. The main outcome variable is Lung function at 3 and 6 months. Frequencies with percentages will be reported for categorical variables like lung functions, gender, fever at the onset of illness, history of influenza vaccine, category of illness, co-morbid, ICU stay, travel history, smoking status, mechanical ventilation etc. For quantitative variables such as age (years), weight (kg), height (cm), length of hospital stay (days), length of ICU stay (days), duration of mechanical ventilation (days), FEV1, FVC, FEV1/FV ratio, qSOFA score, CURB-65 score, mean/median ± standard deviation/IQR will be reported depending upon the data distribution. Chi-square/fischer exact test will be applied for assessing the association between two categorical variables and student-*t*-test/Mann-Whitney U test for the two quantitative variables.

#### Study Progress

Recruitment to the Lung function COVID-19 study began in August 2020 and is currently ongoing. The study is expected to conclude in March 2021. The current protocol is version 2.0 and is dated 7 July 2020.

## Discussion

There are several strengths and limitations of the study. Recruitment will be using a prospective approach which will be helpful in monitoring the course of the disease in real time and plan and prescribe any intervention as needed. The variables in the study have been selected after thorough literature search and opinion of the experts in the field of infectious diseases. The study team includes a mix of researchers, physicians trained in the field of infectious diseases, coordinators and field staff trained in managing mega projects by maintaining quality standards of data management.

The findings will play crucial role in the development of programs for survivors at population level. The study findings will be disseminated to stakeholders including; community, government and academia. Moreover, strategies to avert consequences of disease such as a rehabilitation programs could be devised based on the study findings.

This is a single center study with a small sample size. The current sample size may not be the representative of population at large however; the study has strong potential to develop evidence on the current state of lung function among survivors. Follow-up at 6-months could be a challenge with the unpredictable pandemic situation.

## Ethics Statement

The studies involving human participants were reviewed and approved by Ethical Review Committee, Aga Khan University, Karachi, Pakistan. Written informed consent to participate in this study was provided by the participants' legal guardian/next of kin.

## Author Contributions

SQ and FQ conceptualized the study design and methodology. NN and NR provided input as an expert in infectious diseases and pulmonology. FQ, SQ, NA, and ZH contributed in the write up of manuscript. All authors have reviewed and agreed to publication.

## Funding

This study was funded through a grant from the Bill and Melinda Gates Foundation, grant no: (OPP1176356). The funders had no role in the study design.

## Conflict of Interest

The authors declare that the research was conducted in the absence of any commercial or financial relationships that could be construed as a potential conflict of interest.

## Publisher's Note

All claims expressed in this article are solely those of the authors and do not necessarily represent those of their affiliated organizations, or those of the publisher, the editors and the reviewers. Any product that may be evaluated in this article, or claim that may be made by its manufacturer, is not guaranteed or endorsed by the publisher.

## Abbreviations

AKUH: Aga Khan University Hospital
RT-PCR: Reverse Transcriptase Polymerase Chain Reaction
DLCO: Diffusion Lung capacity for Carbon Monoxide
ID: Infectious Disease
ECHO: Echocardiogram
ERC: Ethical Review Committee
SARS-CoV-2: Severe Acute Respiratory Syndrome Coronavirus 2
WHO: World Health Organization
MERS-CoV: Middle East Respiratory Syndrome Coronavirus
SOPs: Standard Operating Procedures
ARDS: Acute Respiratory Distress Syndrome
CT: Computed Tomography
COPD: Chronic Obstructive Pulmonary Disease
ATS: American Thoracic Society
qSOFA: quick Sequential Organ Failure Assessment
ICU: Intensive Care Unit
HDU: High Dependency Unit.
